# A Battery-Less Wireless Respiratory Sensor Using Micro-Machined Thin-Film Piezoelectric Resonators

**DOI:** 10.3390/mi12040363

**Published:** 2021-03-27

**Authors:** Sina Moradian, Parvin Akhkandi, Junyi Huang, Xun Gong, Reza Abdolvand

**Affiliations:** Department of Electrical and Computer Engineering, University of Central Florida, Orlando, FL 32828, USA; parvin.akhkandi@knights.ucf.edu (P.A.); jensenhuang0@Knights.ucf.edu (J.H.); xun.gong@ucf.edu (X.G.); REZA.ABDOLVAND@ucf.edu (R.A.)

**Keywords:** wearable, MEMS, respiratory, wireless, RFID, piezoelectric, RF MEMS

## Abstract

In this work, we present a battery-less wireless Micro-Electro-Mechanical (MEMS)-based respiration sensor capable of measuring the respiration profile of a human subject from up to 2 m distance from the transceiver unit for a mean excitation power of 80 µW and a measured SNR of 124.8 dB at 0.5 m measurement distance. The sensor with a footprint of ~10 cm^2^ is designed to be inexpensive, maximize user mobility, and cater to applications where disposability is desirable to minimize the sanitation burden. The sensing system is composed of a custom UHF RFID antenna, a low-loss piezoelectric MEMS resonator with two modes within the frequency range of interest, and a base transceiver unit. The difference in temperature and moisture content of inhaled and exhaled air modulates the resonance frequency of the MEMS resonator which in turn is used to monitor respiration. To detect changes in the resonance frequency of the MEMS devices, the sensor is excited by a pulsed sinusoidal signal received through an external antenna directly coupled to the device. The signal reflected from the device through the antenna is then analyzed via Fast Fourier Transform (FFT) to extract and monitor the resonance frequency of the resonator. By tracking the resonance frequency over time, the respiration profile of a patient is tracked. A compensation method for the removal of motion-induced artifacts and drift is proposed and implemented using the difference in the resonance frequency of two resonance modes of the same resonator.

## 1. Introduction

It is estimated that from the hundreds of millions burdened with chronic respiratory conditions globally, annually four million die prematurely [1]. This immense humanitarian and economic toll can be alleviated with proper care and monitoring of respiratory function in at-risk patients [1,2].

Respiration rate is an excellent predictor for the need to admit patients at risk of cardiac arrest into intensive care units [3]. Studies suggest that the relative changes in respiration rate are a more significant indicator of at-risk patients than systolic blood pressure in the case of physiologically unstable patients [4]. Regardless of the accuracy of diagnostic tools, limited information gathering at in-clinic visits provides a narrow snapshot of the patient’s physiology that is not measured under natural conditions [5]. This creates an inherent limitation in the quality of the clinical evaluation based on the results of diagnostic tools.

In addition to diagnostic applications, studies have shown the benefit of long-term continuous respiration monitoring for patient’s recovery [6]. As such, research results emphasize the need for respiration monitoring systems that are capable of continuously measuring both respiration rate and respiration depth [7].

Despite the stated benefits of monitoring the respiratory rate, research shows that, even in cases where the patient’s main complication is a respiratory condition, proper measurement is routinely neglected [8]. This could be the result of the relative complexity of conventional respiratory monitoring diagnostic tools that necessitate the patient be connected to a stationary measurement unit with cables and tubes. This cumbersome setup renders continuous long-term monitoring impractical for most patients.

Monitoring systems designed to fill this gap in healthcare can be categorized according to the physiological signatures that are monitored in proxy of respiration [9]. The main categories are airflow temperature [10], moisture content [11], flow rate [12], acoustic signature [13], and chest movement [2]. While these sensors lend themselves to non-intrusive continuous long-term monitoring, typically their on-sensor circuitry and batteries increase sensor unit cost, footprint, and patient discomfort and hinder continuous long-term monitoring.

To address the aforementioned needs in our previous work, a resonant wireless respiration monitoring sensor capable of measuring respiration rate without the need for onboard batteries or circuits was demonstrated for the first time [14]. With a weight of only 9 g, the sensor is composed of a Micro-Electro-Mechanical (MEMS) resonator connected to a commercial RFID antenna operating in the ISM band (@902 MHz) and was wirelessly energized from a transmitter.

Although this sensor offered a compact form-factor (~9 cm^2^), negligible thickness, and low unit cost, the relatively limited measurement range (~25 cm) was less than desired. Here, we build on this work by significantly improving the measurement range from a maximum of 25 to 2 m in this paper, enhancing resilience to motion-induced artifacts, and improving SNR and thus sensor accuracy without sacrificing the sensor’s small footprint and unit cost. To achieve this, we utilize a dual-mode low-loss, high Q, MEMS resonator with a custom-designed RFID antenna. We illustrated that our sensor is capable of wirelessly measuring human respiratory profiles by proxy of moisture condensation and heat exchange.

The structure of this work is as follows. After a brief overview of the operation of the sensing system, we introduce individual components of the sensor, the impact of exposure to respiratory flow on the MEMS resonator as it relates to sensing, and the details of wireless power delivery to the sensor. The results of respiratory monitoring using the proposed sensing system are presented in Section 3. Finally, the main findings of this work are briefly described in the conclusion.

## 2. Systems, Components and Methods

**Figure 1:**
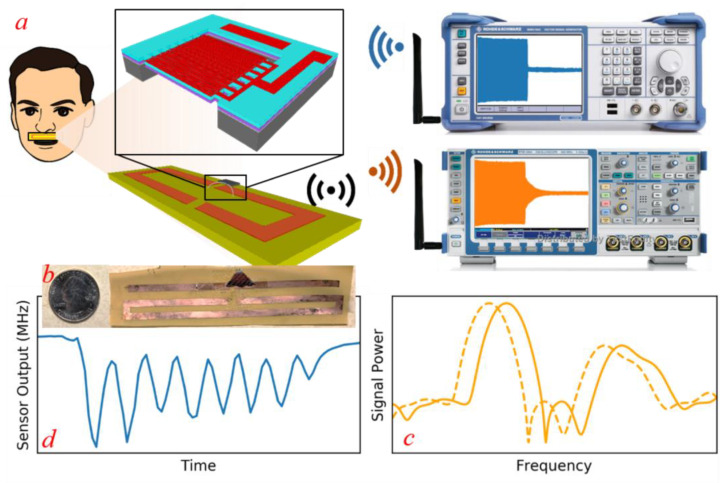
(**a**) A schematic representation of the sensing system. The resonant sensor is excited by a pulsed sinusoidal signal (blue) from a transmitter and its response (orange) as seen by a receiver is analyzed via Fast Fourier Transform (FFT). (**b**) Sample of a sensor used in this study. (**c**) The downward frequency shift of resonators from exposure to respiratory flow. (**d**) Respiratory profile of the healthy subject. Each fall and rise in frequency corresponds to a single respiratory cycle.

The proposed sensor, shown in Figure 1, essentially consists of a piezoelectric-based micro-machined resonator that is excited wirelessly by a pulsed sinusoidal signal transmitted from a transmitter antenna. During the off period of the excitation signal, the resonant sensor returns a decaying sinusoidal signal at the natural resonance frequency of the resonator [15] assuming that the excitation signal frequency was close to the natural resonance frequency of the MEMS resonator. The sensor’s response waveform is recovered by a receiver antenna and transferred to a high-frequency oscilloscope. Similar to the technique used in [15], time gating is utilized to separate the response of the sensor from the excitation signal at the receiver and the resonance frequencies of the sensor are extracted from the response using Fast Fourier Transform (FFT) analysis.

Maximum sensor stimulation occurs when the frequency of the excitation signal matches that of the natural frequencies of the MEMS resonators. As such, using a single signal generator we successively target the resonance frequency of the MEMS resonators. Frequency modulation of the resonance modes stemming from exposure to respiratory flow necessitates that the excitation signal frequency closely follows the resonance frequencies [16]. For this reason, a dynamic frequency locking mechanism is incorporated into the signal acquisition/generation software.

In the following section, we detail how exposure to respiratory flow impacts the MEMS device and we describe the criteria used to design and optimize these devices for passive wireless sensing.

### 2.1. Low-Loss, High-Quality-Factor Piezoelectric MEMS Resonator

The first rendition of the sensor in this work is made up of two RF MEMS resonators wire bonded to a custom-designed flexible dipole antenna. As will be detailed in the following sections, the goal of this multi-resonator operation is the elimination of the environmental effect (such as temperature) on the sensor.

Here, we utilize thin-film piezoelectric-on-substrate (TPoS) MEMS resonators operating near the ISM band (902–928 MHz). TPoS MEMS resonators were chosen based on their low insertion loss and high quality-factor [17]. The MEMS used here, schematically shown in Figure 1, is composed of a piezoelectric layer sandwiched between two metal layers stacked on top of a nanocrystalline diamond substrate. AlN with a thickness of 500nm forms the piezoelectric layer and the nanocrystalline substrate has a thickness of 3 µm. Details of the operation of the MEMS device, including the design and fabrication process, can be found in [18,19].

To model the sensor, an equivalent circuit for the MEMS resonator is developed. Here, the well-established modified Butterworth-Van Dyke (mBVD) model is used [20], which models the effective mass, stiffness, and loss of a single resonance mode as a series inductor ($L_{m})$, capacitor ($C_{m})$, and resistor ($R_{m})$, respectively. In addition, the energy stored in the electrical field formed between the ground and signal electrodes is represented as a capacitor ($C_{0})$ parallel with the series RLC mechanical branch. Furthermore, a resistor modeling ohmic losses of the resonator is placed in series with the other elements. The antenna was modeled as a complex load in series with a voltage source in transmission and receiving mode, respectively.

**Figure 2:**
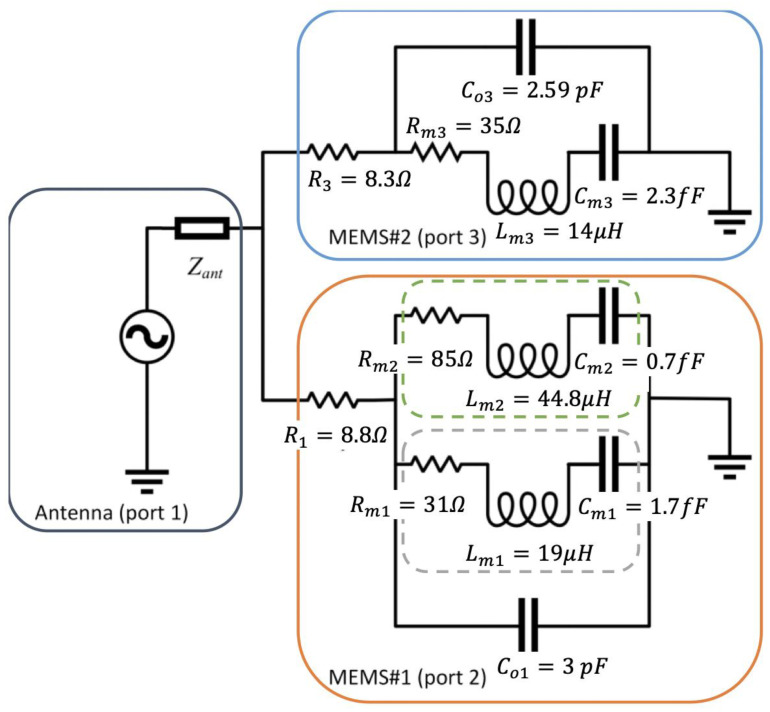
The equivalent circuit model of the sensor in receive mode. The model of the sensor (MEMS#1) is outlined in orange with the mechanical branches corresponding to each of the two modes outlined in green and gray. The model of the reference device (MEMS#2) is outlined in light blue. The antenna is modeled as a voltage source in series with a complex load is outlined in dark blue.

The complete model of the sensor including the antenna and the calculated mBVD model values for the two MEMS resonators used in this paper is shown in Figure 2. Notice that MEMS#1 has two series RLC branches with each corresponding to a different resonance mode.

**Figure 3:**
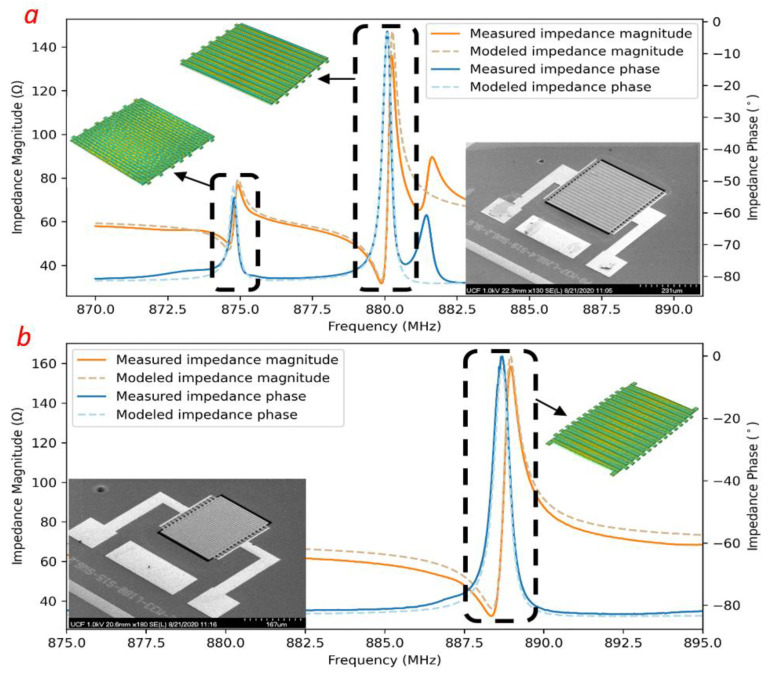
The measured and modeled impedance magnitude and phase of MEMS#1 (**a**) and MEMS#2 (**b**) resonators used in the sensor with the mode shapes (as simulated using COMSOL) corresponding to (**a**) 874.7 MHz and 879.9 MHz and (**b**) 888 MHz modes placed as insets.

The impedance responses of the two resonators intended to be used for sensing are shown in Figure 3. The modes of interest were measured to be at 874.7 MHz, 879.9 MHz, and 888 MHz with an unloaded quality factor of ~1212, ~2750, and ~1750, respectively. The proximity of the resonance frequencies of the two MEMS devices assists in the design of a dipole antenna with acceptable efficiency at both frequencies. However, if the resonance frequencies are too close, we risk misidentification of the modes during measurement.

The impedances of the two resonators, as modeled by the mBVD model (shown in Figure 2), were plotted alongside the measured impedance and are shown to be in good agreement.

### 2.2. Sensing Mechanism

The response of the sensor to respiratory flow can be thought of as a reaction to the modulation of the MEMS resonators’ temperature and surface vapor condensation due to the periodic expulsion of air from the lungs [9]. Through disparate coupling pathways, both surface condensation and increase in temperature result in a reversible drop in MEMS device’s resonance frequency. Critical for our application, however, the resulting frequency shift due to both mechanisms is of the same polarity (negative).

Heat transferred from hot exhalatory flow to the resonator has the principal effect of lowering the effective stiffness of the resonator which in turn decreases the resonance frequency [19]. The temperature-frequency characteristic of an MEMS resonator is dependent on the properties of the material that constitutes the resonators. For a given respiration-induced temperature change of ΔT, the frequency shift can be calculated as:

(1) $$\Delta f_{T}=TCF\times f_{0}\times \Delta T$$

Here, TCF is the temperature coefficient of frequency and has been measured to be effectively constant (~−9.6 ppm) in the temperature range of interest for the targeted lateral extensional resonance mode of the TPoS MEMS resonators [18] used here, and $f_{0}$ is the initial resonance frequency of the same mode.

**Figure 4:**
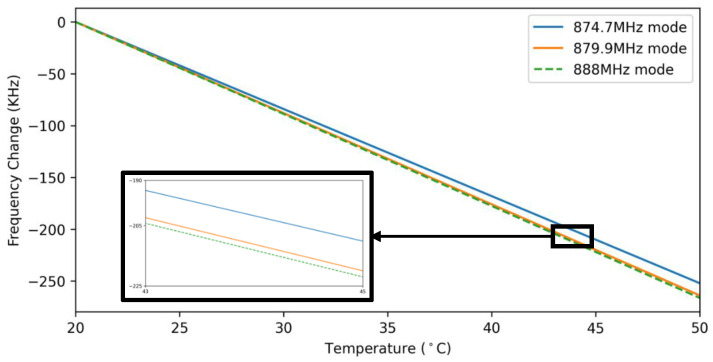
Simulated temperature-frequency profile of 874.7 and 879.9 MHz modes of thin-film piezoelectric-on-substrate (TPoS) MEMS resonator used in the sensor. This resonator is fabricated on a 3 µm nanocrystalline diamond-on-silicon substrate covered by 500 nm of sputtered AlN. The frequency change profile of the 879.9 and 888 MHz modes is very similar because of their common operating mode, and the small variation can be attributed to the difference in center frequency.

The frequency shift as a function of temperature was simulated using COMSOL for all three modes measured in Figure 2. The resonance frequency for a given temperature is simulated from eigenmode analysis. To achieve this, first, the Temperature Coefficients of Elasticity (TCE) of the AlN and Mo layers were extracted from [21,22], respectively. Next, using these values we extract the TCE of the resonators’ nanocrystalline diamond layer by fitting the simulated temperature-frequency profile to the measured profile. Knowing this value, we can simulate the temperature-frequency profile of all 3 modes, as shown in Figure 4.

As exhaled air with high moisture concentration passes over the MEMS resonator, a thin layer of water vapor condenses on the surface of the device. This thin layer increases the effective mass of the resonator proportional to its coverage and thickness. Modeling an MEMS resonator as a mass-spring-damper system, the resonance frequency can be calculated as:

(2) $$f_{0}=\frac{1}{2\pi }\sqrt{\frac{k}{m_{0}}}$$

Here, $m_{0}$ is the initial effective mass and k is effective stiffness. Assuming a small mass loading term (Δm), the frequency shift is calculated to be proportional to the effective mass of the adsorbed water vapor:

(3) $$\Delta f_{m}=-\frac{f_{0}}{2m_{0}}\Delta m$$

In addition to mass loading, adsorption of moisture results in higher acoustic energy loss stemming from both acoustic mismatch between condensation and the micromechanical structure and also high acoustic loss in the condensation layer [23,24].

Both changes in temperature and vapor mass loading are periodic time-varying parameters that are coupled through heat transfer occurring during vapor condensation and evaporation. Therefore, the total sensor frequency shift corresponding to a single resonance mode resulting from exposure to respiratory flow can be written as:

(4) $$\Delta f\left(t\right)=-\frac{f_{0}}{2m_{0}}\Delta m\left(t\right)-TCF\times f_{0}\times \Delta T\left(t\right)$$

The same sensing pathways that facilitate respiration sensing can also act as a source of measurement error considering environmental variability. As described in Equations (3) and (4) (and illustrated in Figure 4), any change in temperature, including environmental temperature, results in a change in the resonance frequency of the resonators. If this change is sudden (such as in the case of exposure to sunlight and opening of refrigerator door), it can be incorrectly categorized as a respiration event. As shown in Figure 4, the temperature-frequency profile of the 879.9 and 888 MHz modes are almost identical in the 20–50 °C range. In effect, by monitoring the difference in resonance frequencies we can distinguish frequency change stemming from environmental temperature variation from respiration.

(5) $$\Delta f_{888}\left(t\right)-\Delta f_{879.9}\left(t\right)={\left[-\frac{f_{0}}{2m_{0}}\Delta m\left(t\right)\right]}_{888}-{\left[-\frac{f_{0}}{2m_{0}}\Delta m\left(t\right)\right]}_{879.9}$$

The RFID antenna exhibits slightly different impedance characteristics as a function of environmental electromagnetic loading. As a result, the sensor is susceptible to motion-induced artifacts stemming from spatial and temporal changes in surrounding objects. The change in sensor frequency resulting from motional artifacts is expected to be similar for different modes; therefore, by monitoring the difference in the resonance frequencies of two different modes, we can discern respiration from such artifacts. The viability of the two compensation methods is studied experimentally in Section 3 of this work.

### 2.3. Antenna Design

A wearable wireless sensor that is designed to enable patient mobility requires a compact, lightweight, and omnidirectional antenna. While many commercial RFID antennas meet the above criteria, they are specifically designed for certain RFID tag chips and therefore cannot be used for our MEMS sensors. It should be noted that the impedance mismatch between the MEMS sensors and antenna is a major source of efficiency loss and thus must be kept at a minimum.

While it is feasible to incorporate an on-sensor matching network, this additional component would both increase sensor loss and complicate the development of the sensor. To this end, we developed a custom dipole antenna with lateral dimensions of ~2 × 6.5 cm^2^, negligible thickness, and a weight of only ~10 g. Based on [25], this simple uniplanar antenna was designed specifically for RFID tag applications and has the advantage of relatively straightforward impedance tunability whilst retaining a compact design.

To design the antenna, first, the S-parameters of the MEMS resonators were characterized using Rhode & Schwarz ZNB-8 Vector Network Analyzer (VNA). Given that the two MEMS devices are electrically connected in parallel, Keysight Advanced Design System (ADS) was used to calculate the equivalent impedance.

The major challenge in designing this antenna is achieving acceptable impedance matching between the antenna and MEMS sensor despite the difference in impedance at their respective resonance frequencies. Rather than matching the antenna perfectly at only one of the MEMS resonance modes, our solution was to match the regression line of the equivalent MEMS sensor’s impedance at the resonance modes. Whilst the power lost to impedance mismatch is not eliminated at either of the MEMS sensor’s resonance modes, this design achieves the overall minimum impedance mismatch. It should be noted that this antenna was originally designed with only 879.9 and 880 MHz modes in mind.

**Figure 5:**
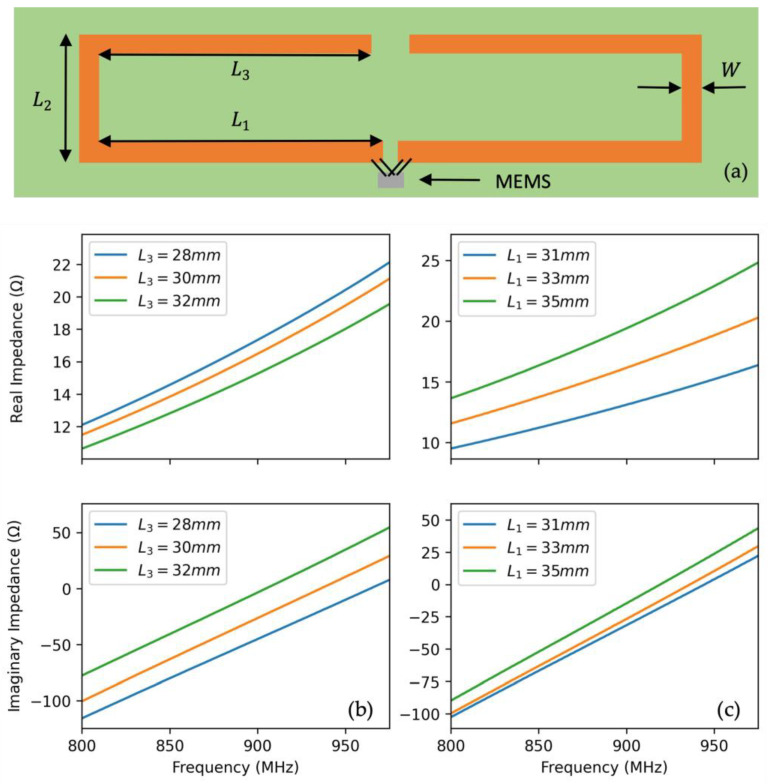
(**a**) Schematic of the sensor with the critical design dimensions of the RFID antenna highlighted. (**b**) Simulated antenna impedance for $L_{3}\;$ sweep and W = 3 mm, $L_{1}\;$ = 33 mm, and $L_{2}\;$ = 33 mm. (**c**) Simulated antenna impedance for $L_{1}\;$ sweep and W = 3 mm, $L_{2\;}$ = 33 mm, and $L_{3}\;$ = 30 mm.

To realize this design, first, the antenna dimensions are estimated such that the effective length of the antenna is equivalent to a half wavelength at the antenna’s center frequency. In the next step, ANSYS High-Frequency Structure Simulator (HFSS) is utilized to fine-tune the dimensions to achieve matching to the equivalent impedance of the two MEMS. This fine-tuning process is illustrated in the HFSS simulation results shown in Figure 5. We observed that the real and imaginary parts of the antenna impedance are mainly a function of $L_{1}$ and $L_{3}$, respectively.

**Figure 6:**
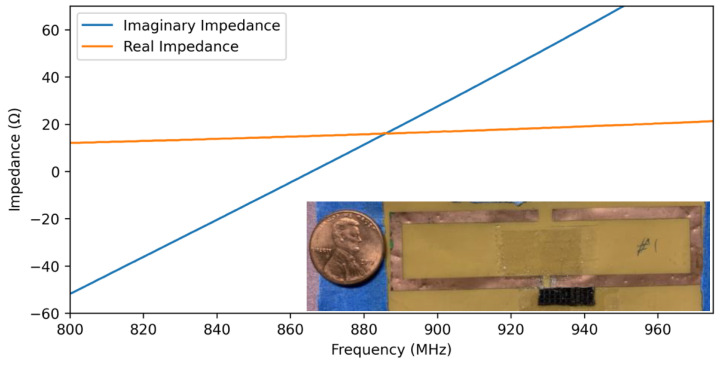
Real and imaginary impedances of the final antenna design with the fabricated antenna inserted as inset.

The impedance profile of the finalized design with dimensions of W = 3 mm, $L_{1}$ = 35 mm, $L_{2}\;$ = 20 mm, and $L_{3}\;$ = 37.2 mm is shown in Figure 6. Using this design, the power transfer efficiency between the antenna and 3 MEMS resonance modes is shown in Table 1. The efficiency was found to be noticeably higher for the 879.9 and 888 MHz modes at 77.6% and 81.7%, respectively, as compared to the 874.7 MHz mode at 20%. This difference can be mainly attributed to the significant difference in impedance at 874.7 MHz compared to the other two modes.

The antenna was fabricated using the print-screen method [25] on a flexible Pyralux copper laminate sheet with a copper and polyimide thickness of 35 and 25 µm, respectively. In the first step of fabrication, the paper mask is printed and laminated onto the Pyralux sheet by applying heat. Next, the sheet is placed in a copper etchant bath for ~4 min.

### 2.4. Power Delivery to Sensor

Power delivery is a fundamental limitation of passive sensing. In our respiratory sensor, the distance between the patient and transceiver unit, and hence the quality of the mobility offered to the patient, is directly limited by the strength of the signal delivered to and received from the sensor. As such, understanding the relationship between sensing distance and wireless signal strength is crucial. For this reason, we have developed a framework that connects the transmitted power to antenna characteristics, sensor-transceiver distance, sensor characteristics, signal acquisition settings, and ultimately the strength of the sensor’s signal, as measured by the receiving unit.

For a given excitation signal, the power delivered to the passive wireless sensor is determined by the antenna gain, sensor distance, and sensor loss and can be calculated using the Friis Equation [26].

(6) $$\frac{P_{S}}{P_{Tx}}=\frac{G_{S}G_{Tx}\lambda^{2}L_{S}}{{\left(4\pi \right)}^{2}D_{TS}^{2}}$$

Here, $P_{S}$ and $P_{Tx}$ are the power of the signal delivered to the sensor and transmitted by the transmitter antenna, respectively, and $G_{Tx}$ and $G_{S}$ are the gain of the transmitter and sensor antenna, respectively. $D_{TS}$ represents the transmitter-sensor distance. λ is the EM wavelength at the excitation signal frequency (assuming the frequency of excitation signal matches either MEMS resonance mode) and $L_{s}$ represents the total power lost in the sensor. Sources of loss include impedance mismatch between the resonator and antenna, Ohmic loss, dielectric loss, and losses occurring in the MEMS resonator [27]. Modifying the Friis equation for ringdown sensor measured using FFT windowing, the power of the sensor’s response as received by the oscilloscope can be calculated as:

(7) $$\frac{P_{Rx}}{P_{Tx}}=\frac{G_{Rx}G_{Tx}G_{S}^{2}\lambda^{4}L_{S}^{2}}{{\left(4\pi \right)}^{4}D_{RS}^{2}D_{TS}^{2}}\times \frac{\left(1-e^{-T_{w}\omega /Q}\right)}{T_{w}\omega /Q}$$

Here, $T_{w}$ is the FFT time window, ω is the angular frequency of the MEMS resonance mode, and Q is the effective quality factor at the receiver, as measured by the ringdown method. It should be noted that while theoretically increasing the transmitted power can significantly improve measurement range, in practice transmitted power is often limited by the RF front end (amplifier linearity and noise figure) and also concerns patient safety from prolonged exposure to high-power RF radiation. This highlights the importance of minimizing sources of energy loss in the sensor to maximize measurement distance. This equation is applied to the respiratory sensor in the following section and is utilized to calculate the actual total power loss in the sensor after all the different components are integrated.

## 3. Results and Discussion

**Figure 7:**
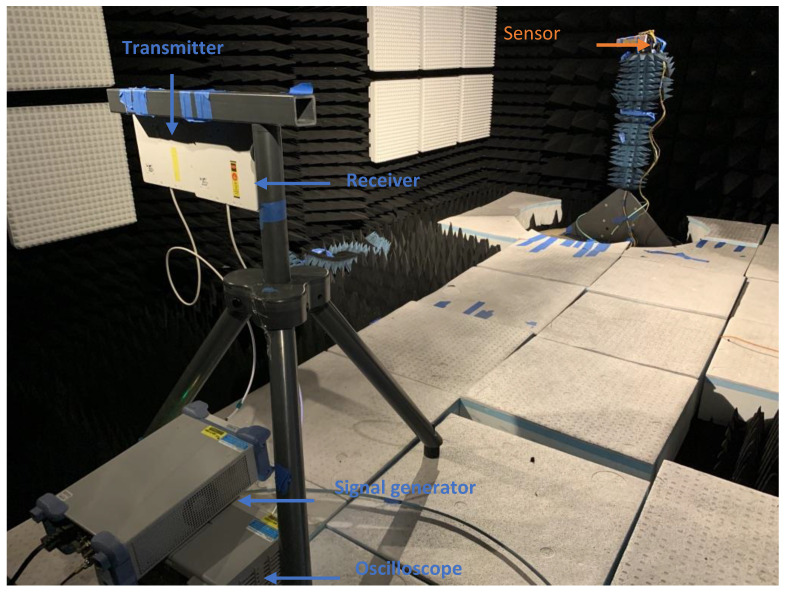
Measurement setup in an anechoic chamber for studying power at the receiver end as a function of sensor distance. Both the Signal generator and oscilloscope are controlled via LabVIEW from a PC placed outside the chamber.

**Figure 8:**
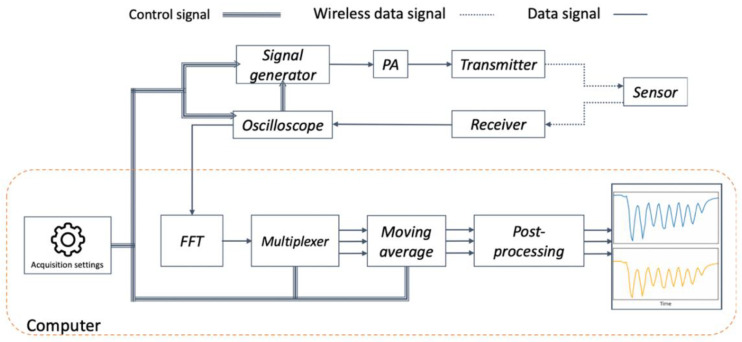
Signal acquisition flow chart highlighting the major components/stages of the respiratory sensing system. The Power Amplifier (PA) component is strictly optional. Here, the profile derived from the reference MEMS is not displayed in the output.

The measurement setup and the acquisition flow chart used here are shown in Figure 7 and Figure 8, respectively. The transmitter and receiver antennas were connected to a Rohde & Schwarz, Munich, Germany, SMC100A signal generator and RTO 1024 oscilloscope, respectively. The signal generator is externally triggered by the oscilloscope and both are controlled and recorded through their LAN port from a remote PC unit using National Instruments, Austin, TX, USA, LabVIEW. The excitation signal alternatively targets the frequency of the resonance modes and their response in the form of a decaying sinusoidal signal is recorded by the oscilloscope and subsequently passed on to the PC for FFT analysis.

The high quality-factor of the MEMS resonance modes combined with the frequency-modulated sensing mechanism necessitates a dynamic frequency tracking system whereby the frequency of the excitation signal locks onto that of the resonance modes throughout the measurement. For this purpose, the frequency of the excitation signal targeting a given mode was set as the moving average of the preceding measured resonance frequencies of that mode.

In sensing applications, typically the size of the moving average window is determined as a compromise between responsiveness and SNR; however, in this case, given that a slow response to shifts in resonance frequency results in lower SNR, a small window is preferred. A moving average with a larger window is applied in the post-processing step. The window size for the tracking system and post-processing step was 2 and 8, respectively.

The time resolution (sampling rate) of the respiratory profile determines how faithfully the patient’s respiration is reconstructed and is a critical parameter as it sets the maximum detectable feature frequency through the Nyquist–Shannon sampling theorem. Given that the typical respiratory cycle of an adult human lasts ~4 s, our measurement system’s time resolution of ~130 ms fulfills the Nyquist frequency condition and can theoretically resolve features up to ~3.85 Hz. It should be noted that, in our measurement system, the transfer of signals acquired by the oscilloscope to the PC and their processing by LabVIEW is the determining factor for time resolution.

### 3.1. Resilience to Environmental Interference

The goal of incorporating more than one MEMS resonator into the sensor was to distinguish between the effects of respiratory flow and environmental variability on the sensor. The two major sources of environmental variability focused in this work are (1) discerning changes stemming from respiratory flow from environmental temperature variation and (2) negating motional artifacts stemming from small changes in the performance of the antenna as a function of spatial and temporal variability in electromagnetic environmental loading.

Given that a MEMS resonator’s resonance frequency is a function of temperature, any sudden change in environmental temperature can be miscategorized as a respiratory event. As such, by incorporating two MEMS devices with similar TCF (as shown in Figure 4), we sought to remove frequency modulation originating from environmental temperature change from the respiratory profile. In other words, we endeavored to decouple temperature-induced frequency shift from mass loading frequency shift.

**Figure 9:**
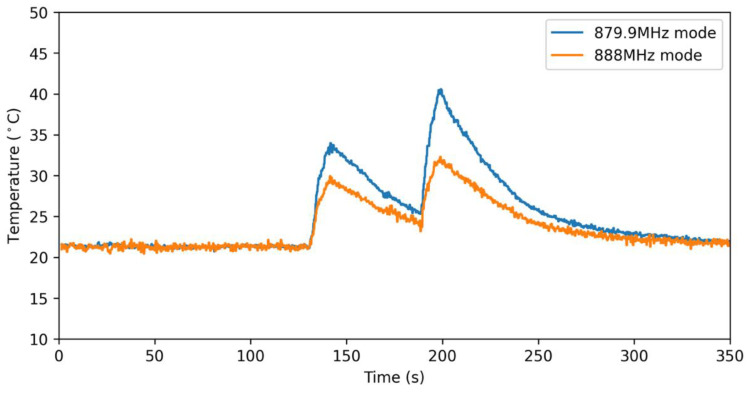
Measured temperature shift in 879.9 and 888 MHz modes in response to two separate heating periods. Before the first heating event, the temperature of the resonators (each correlating to an individual mode) was identical, and, following the second cool-down period, the temperature of the modes converged.

The viability of this decoupling mechanism was experimentally studied by flowing hot air over the sensor and monitoring the frequency shift. This experiment is designed to replicate a sudden change in environmental temperature in the absence of any significant mass loading. As shown in Figure 9, the frequency shift exhibited by the 879.9 and 888 MHz modes is not consistent with the assumption of equal MEMS resonator temperature that underpins the decoupling mechanism. We hypothesize that this temperature gradient is in part the result of unequal heat transfer due to uncontrolled airflow at the surface of the sensor.

**Figure 10:**
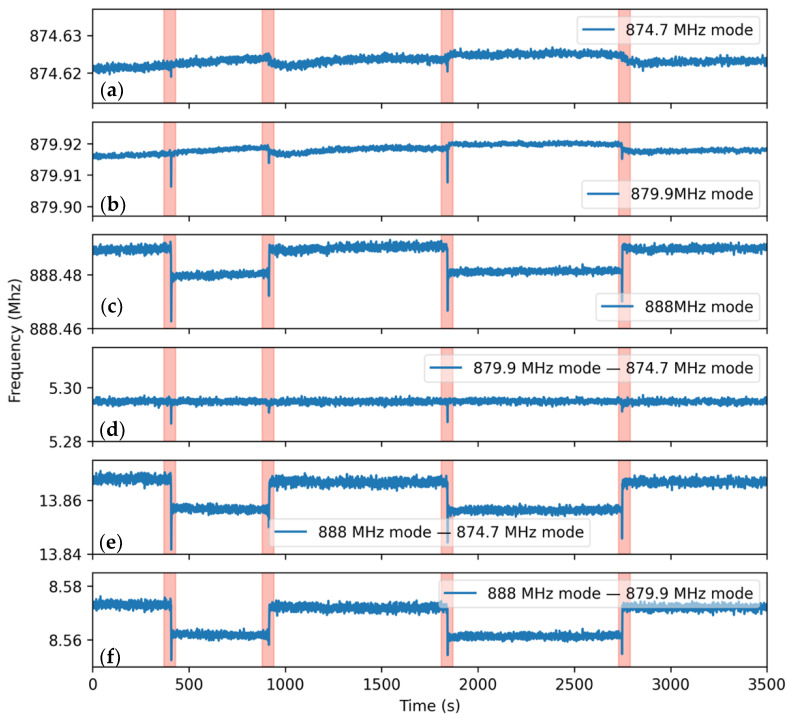
(**a**–**c**) Measured frequency corresponding to each of the sensor’s 3 resonance modes at 50 cm distance and peak and mean powers of 80 mW and 80 µW, respectively. The sensor was rotated by 90° (highlighted in red) to simulate the sudden rotation of the patient’s body. (**d**) For the 874.7 and 879.9 MHz modes, this frequency shift can be canceled by subtracting the 879.9 and 874.7 MHz modes, whilst the same compensation method was found to be ineffective between 888 and 879.9 MHz (**e**), 888 MHz, and 874.7 MHz (**f**).

The RFID antenna exhibits a slightly different impedance profile depending on electromagnetic loading from its environment. The impact of the orientation of the sensor concerning the excitation and receiver antennas was tested by four separate 90° rotations of the sensor. This experiment was designed to replicate the rotation of the patient’s body. The measurement was performed for a peak and mean power of 80 mW and 80 µW at a distance of 50 cm in an anechoic chamber. The results shown in Figure 10 illustrate that sensor orientation significantly impacts the frequency corresponding to both modes, as seen by the receiver antenna.

Without a compensation mechanism, the sensing system would categorize the rotation of a patient’s head as a respiration event. However, as shown in Figure 10, by tracking the difference between the frequencies of the two modes, we can effectively identify changes in frequency associated with motion-induced artifacts. It should be noted that the same compensation mechanism was not observed between either of the two resonance modes of MEMS#1 (874.6 MHz and 879.9 MHz) and that of MEMS#2 (888 MHz). The spike-like drop in frequencies is due to the physical handling of the sensor by the experimenter’s hand.

Given the results of the two experiments, we concluded that while the sensor is capable of motion-induced artifact removal, in its current format, distinguishing rapid changes in environmental temperature from the respiratory flow is infeasible without utilizing a software-based solution. As a result, the 888 MHz resonance mode was not monitored in the following measurement results.

**Figure 11:**
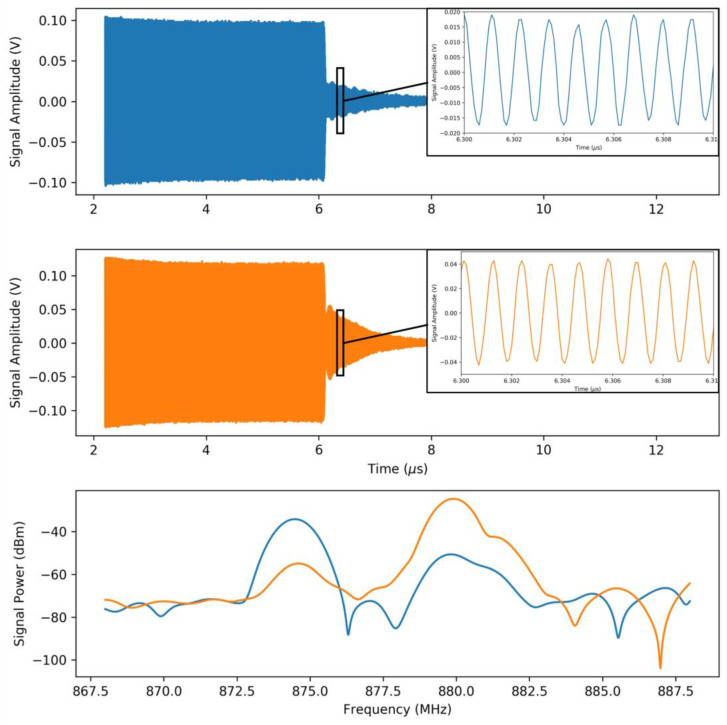
Excitation signal and sensor response, as measured by the receiving antenna, for the 874.7 (top) and 879.7 MHz (middle) modes. The FFT of the two signals are superimposed and shown in the bottom panel.

Using the measurement setup described above, the respiratory profile of a healthy adult was measured with a mean transmitted power of 80 µW and peak transmitted power of 80 mW. The response of the sensor, as seen by the receiver antenna, is shown in Figure 11.

**Figure 12:**
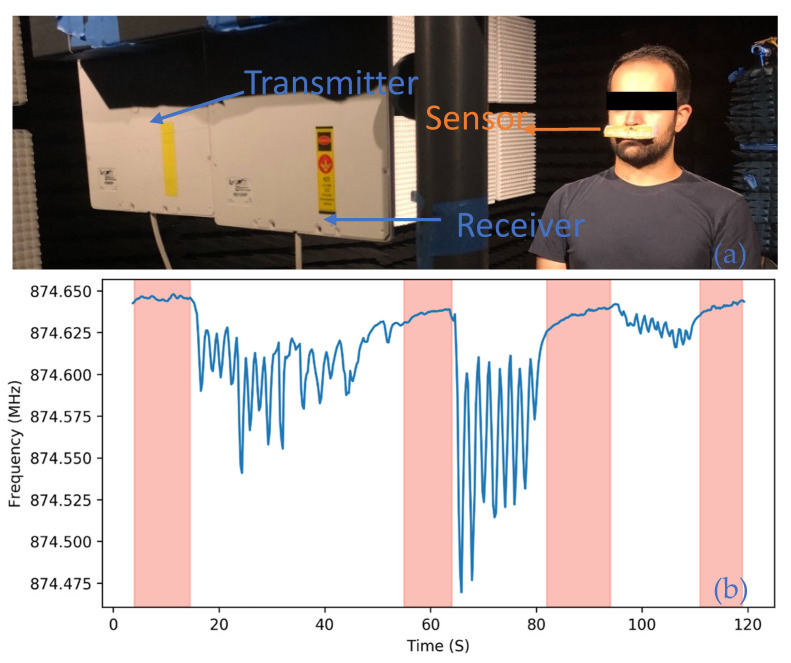
(**a**) Human respiratory measurement setup, illustrating the sensor placed on the patient’s upper lip region. (**b**) Respiratory profile of a human subject demonstrating non-uniform, uniform moderate, and shallow respiration in that order. Each drop in the frequency and subsequent recovery constitutes a single exhalation and inhalation cycle. Periods in which the patient ceased to breathe are highlighted in red.

As shown in Figure 12, the sensor has measured three distinct respiratory patterns, irregular, uniform-normal, and shallow, each of which is interrupted by a brief period in which the patient ceased breathing. This pattern of brief cessation of breathing events is commonly found in patients suffering from sleep apnea.

### 3.2. Power Delivered to the Sensor

**Figure 13:**
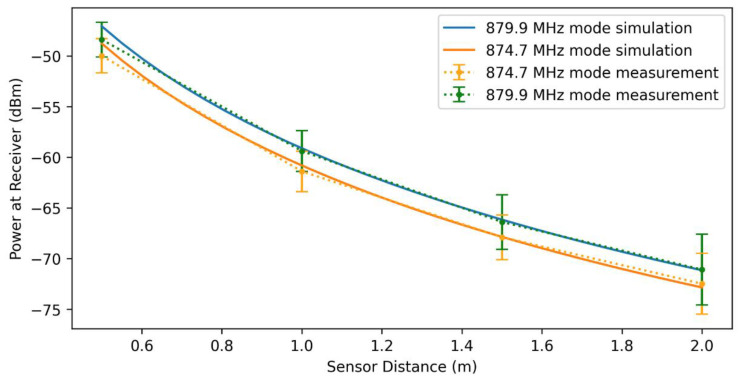
Signal power at receiver for a peak transmitter power of 19 dBm. The difference in measured and simulated values can be largely attributed to the impact of the directionality of the transmitter and receiver antennae at 50 cm.

The response of the sensor at the 874.7 and 879.9 MHz modes were measured as a function of distance from the base unit for a peak and mean transmitted power of 80 mW and 80 µW, respectively. By knowing the performance of the sensor for a given input power, we can use the modified Friis equation, Equation (7), for sensor, transmitter, and receiver antenna gains of 1.4 dBi, 8 dBi, and 8 dBi, respectively, to predict signal strength as a function of sensor distance. The simulated and measured signal power is shown in Figure 13.

By fitting Equation (5) to the measured power at the receiver, the total effective loss of the sensor for the 874.7 and 879.9 MHz modes was found to be ~15 and ~13.4 dB, respectively. The difference between these values and the simulation results in Table 1 can be attributed to discrepancies between HFSS simulation of the antenna and the fabricated antenna, wire-bond inductance, and degradation of MEMS resonator due to aging effects.

**Figure 14:**
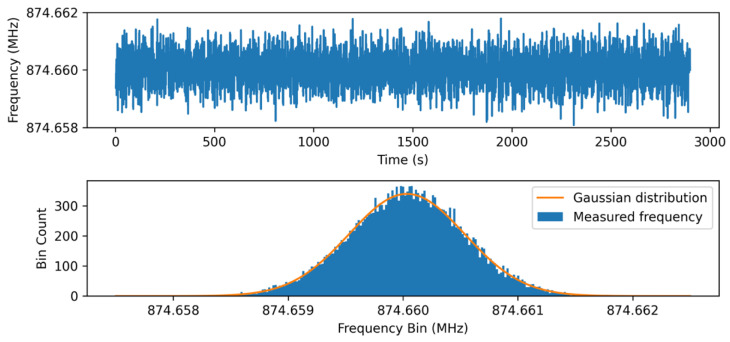
**Top**: Sensor measurement for 45 min for a peak transmitted power of 80 mW and mean transmitted power of 80 µW. **Bottom**: Histogram of sensor response fitted with Gaussian distribution with a standard deviation of ~503 Hz. Each count corresponds to a single measured response.

The precision of the sensing system was evaluated by calculating the measurement variance for a relatively long duration (~45 min) at a distance of 0.5 m from the receiver and excitation antenna in an anechoic chamber. Similar to the previous experiment, the peak and mean transmitted power were 80 mW and 80 µW, respectively. Following measurement, frequency drift stemming from environmental temperature variation was removed in post-processing. As shown in Figure 14, by plotting the histogram of the measurement and fitting it to a normal distribution, the standard deviation was calculated to be ~503 Hz. Using this value, we can calculate the SNR = μ/σ to be 124.8 dB.

## 4. Conclusions

A passive wireless respiratory sensing system based on the frequency modulation of a TPoS MEMS resonator was developed. With a size of only ~10 cm^2^, the sensor was shown to be capable of measuring a respiratory profile from up to a distance of 2 m from the transceiver unit for mean transmitted power of 80 µW and peak transmitted power of 80 mW. The SNR was measured to be 124.8 dB at a 50 cm sensor distance with the same transmitted power.

The respiratory sensor is composed of an antenna wire bonded to two low-loss piezoelectric MEMS resonators and is excited via a transmitter antenna and its response is recorded by a second receiver antenna. We showed that an increase in temperature and mass loading from surface vapor condensation, both stemming from exposure to exhaled airflow, results in a substantial and reversible drop in the MEMS resonance frequencies. FFT analysis is then used to extract the shift in MEMS devices’ resonance frequencies and is plotted over time to construct the patient’s respiratory profile.

To minimize energy lost to impedance mismatch between the MEMS resonators and sensor antenna, a custom RFID antenna was designed to achieve optimal impedance matching with two MEMS resonators. This was achieved despite their significant difference in impedance at their respective resonance modes.

We showed that the Friis equation, widely used to calculate the maximum operating distance for wireless passive devices, is inadequate for ringdown-type sensors. A modified version of the equation, taking into account the FFT window and sensor quality factor, was presented and used to measure the effective loss of the sensor at different operational modes.

The sensor was shown to be capable of clearly distinguishing shallow and irregular breathing from normal respiration and was also utilized to detect brief cessation of breathing events, important for studies of sleep apnea.

The sensing system utilizes a novel method for canceling the effect of motion-induced artifacts and drift on the response of the sensor. It was shown that by incorporating an MEMS resonator with two modes near the frequency of interest and monitoring the difference between their resonance frequencies, frequency shifts stemming from variation in the antenna’s position can be discerned from respiration.

## Figures and Tables

**Table 1:** Simulated power transfer efficiency between the final antenna design and 3 MEMS resonance modes. MEMS1 and MEMS2 are the sensor and reference devices, respectively. MEMS and port numbers are detailed in Figure 4.

Resonance Mode (MHz)	Z_antenna_ (Port 1)	Z_MEMS1_ (Port 2)	Z_MEMS2_ (Port 3)	S11 (dB)	S21 (dB)	S31 (dB)
874.7	15.6 + 5.8j	24.109−44.57j	21.91−24.3j	−3.5	−3.5	−9.6
879.9	15.9 + 9.9j	24.3−19.9j	9.9−60.9j	−11.3	−0.8	−10.5
888	16.4 + 16.9j	9.1−62.4j	21.9−24.3j	−14.1	−10.2	−0.6

## Data Availability

Data sharing not applicable.
